# Development of a sensitive droplet digital PCR according to the HPV infection specificity in Chinese population

**DOI:** 10.1186/s12885-023-11529-3

**Published:** 2023-10-23

**Authors:** Nan Lv, Yue Zhao, Yiying Song, Mingyu Ji, Yunying Zhou

**Affiliations:** 1 Department of Clinical Laboratory Diagnosis, Jinan Central Hospital, Shandong University, Jinan, 250013 People’s Republic of China; 2 Medical Research & Laboratory Diagnostic Center, Central Hospital Affiliated to Shandong First Medical University, No.105, Jiefang Road, Lixia Area, Jinan, 250013 People’s Republic of China

**Keywords:** Human papillomavirus, Infection specificity, Droplet digital PCR, Viral load

## Abstract

HPV16 and 18 are positively correlated with cervical carcinogenesis. However, HPV prevalence tends to vary according to region, nationality, and environment. The most prevalent high-risk (HR) HPV genotypes are HPV16, 52, 58, 56, 18, 33, and 45), while the low-risk (LR) genotypes are HPV6 and 11 in the Chinese population. Importantly, undetectable low-copy HPV DNA could be an important indicator of integration into the human genome and may be a precursor to cancer progression. The HPV viral load changes dramatically, either increasing or decreasing rapidly during carcinogenesis, and traditional quantitative real-time PCR (qPCR) cannot accurately capture this subtle change. Therefore, in this study, a reliable droplet digital PCR (ddPCR) method was developed to simultaneously detect and quantify HPV genotypes. The ddPCR quantitative results showed high accuracy, sensitivity, and specificity compared to qPCR results employing the same clinical specimens and supplemented the ddPCR assay for HPV52/56/58/6 genotypes according to the infection specificity of the Chinese population. In summary, this procedure is valuable for quantifying HPV DNA, especially under conditions of low template copy number in cervical intraepithelial neoplasia (CIN) and/or cervical cancer. Additionally, this method can dynamically observe the prognosis and outcome of HPV infection and thus be used as an effective means for real-time monitoring of tumor load.

**Supplementary Information:**

The online version contains supplementary material available at 10.1186/s12885-023-11529-3.

## Introduction

Cervical cancer is different from other cancers because persistent infection with high-risk human papilloma viruses (HR-HPVs) is a key step in carcinogenesis [1–3]. Non-HPV-related cervical cancer is rare, accounting for less than 1% of all newly diagnosed cases [4]. HPVs are small DNA tumor viruses that exist in an episomal form with a low copy number [5] and promote gene expression changes and carcinogenesis once the HPV DNA is integrated into the host genome [6, 7]. Among the numerous genotypes, HPV16 and 18 have always been considered closely related to 60% of cervical intraepithelial neoplasia (CIN) and 70% of cervical cancer cases [8, 9].

The detection of HPV DNA with a low copy number may be an indicator of tumor transformation, which in turn affects the occurrence and progression of CIN [10]. The current screening test is a cytological staining-based technique and HPV-specific qualitative PCR, which reduces the incidence and mortality of cervical cancer; however, its sensitivity and accuracy are still insufficient to detect low-copy viruses. Therefore, there is an increasing demand for developing more specific HPV detection methods for the screening and monitoring of HPV-related tumors.

Droplet digital PCR (ddPCR) is an innovative technology with improved precision and sensitivity (up to 0.001%) at low template concentrations [11], and has been effectively used for the early screening of tumors [12]. Previously, the detection and quantification of HPV DNA using ddPCR was limited to specific HPV types, including HPV16, 18, 33, 45, 11 [13, 14]. Nevertheless, HPV prevalence tends to vary according to region, nationality, and environment. In the Chinese population, HPV16, 58, 52, 33, and 18 are the most common HPV types causing cervical cancer and precancerous lesions [15]. For example, infection with the HPV58 genotype in the middle and lower reaches of the Yangtze River in the south is significantly higher than that in the north, while HPV58 or 56 genotypes in Hunan Province and Guangzhou City are the third most common genotypes after 16 and 18. Additionally, HPV16, 58/56, 52, and 18 are the main types in West China.

In view of the specificity of HPV infection in the Chinese population and the current methodological limitations, it is necessary to develop a more sensitive and reliable (with respect to accuracy and specificity) ddPCR method that can simultaneously detect and quantify multiple HPV genotypes.

## Materials and methods

### Samples

This study was approved by Jinan Central Hospital Affiliated to Shandong University Ethics Service Committee. Samples from anonymized HPV-positive patients collected from 2020 to 2022 were used. Using the HPV genotyping test (which identifies 24 genotypes), positive samples with a low viral load of the most prevalent 7 high-risk genotypes (including HPV16, 52, 58, 56, 16, 33, 45) and 2 low-risk genotypes (HPV6 and 11) in the Chinese population were selected, as well as positive samples with a high viral load of HPV DNA (> 10^5^ copies/µL) were used for accuracy and sensitivity evaluation. The samples of the remaining 15 HPV genotypes were mixed and named multi-positive control (M-PC) for specificity evaluation, together with negative control (NC) samples. Fifty clinical samples (including 42 specimens from women participating in health examinations and 8 CIN preneoplastic lesion tissues from women who underwent physical treatment) were included for comparison between ddPCR and qPCR.

### HPV genotyping

The samples used for ddPCR optimization were previously HPV genotested by using the Sinochips LINEAR ARRAY® HPV genotyping test to detect 18 high-risk HPV genotypes (HPV16, 18, 26, 31, 33, 35, 39, 45, 51, 52, 53, 56, 58, 59, 66, 68, 73, 82) and 6 low-risk HPV genotypes (HPV6, 11, 42, 43, 44, 84).

#### DNA extraction

DNA was isolated using the DNA Blood, Cell, and Tissue Kit (TIANGEN TIANamp Genomic DNA kit) according to the manufacturer’s instructions. The concentration and purity of DNA were determined using an ultraviolet spectrophotometer (Thermo Fisher, USA). Microbial DNA extracts thus collected were stored at − 20 °C.

### Primers and Probes Designing for Digital Droplet PCR (ddPCR)

The primers and probes were designed in a duplex, combining each HPV genotype (52, 58, 56, 16, 18, 33, 45, 6, and 11) according to the full sequence of the HPV virus available in the NCBI database. The probes are double fluorescent designed, with the 5’ end as a fluorescent dye reporter and the 3’ end as a quencher. To avoid cross-reactivity between different HPV types, primers targeting the E7 gene, a region of the HPV genome with low similarity between HPV types, were used. Gene probes, primer sequences, and amplicon sizes are summarized in Table 1.

**Table 1:** DNA targets according to Gen Bank and nucleotide sequences of primers and probes

Target gene	Primer or probe	Sequence (5’→ 3’ )	Product length	notes
**HPV 52**	***Forward***	GACATGTTAATGCAAACAAGCG	103	
	***Reverse***	TGACGTTACACTTGGGTCAC		
	***Probe***	CAGAGTGTTGGAGACCCCGACC		
**HPV 58**	***Forward***	GGCATGTGGATTTAAACAAAAGG	121	
	***Reverse***	TCTCATGGCGTTGTTACAGG		
	***Probe***	TGGAGACCCCGACGTAGACAAAC		
**HPV 56**	***Forward 1***	TGCATTGTGACAGAAAAAGACG	73	
	***Forward 2***	AAAGCAATTGCMTTGTGACAGA		(M = A or C)
	***Reverse 1***	CTCCAGCACCCCAAACATG		
	***Reverse 2***	GATGTYTGTCTCCAGCACC		(Y = C or T)
	***Probe***	CCCGGTCCAACCATGTGCTATTAG		
**HPV 6**	***Forward***	CGGTTYATAAAGCTAAATTGTACG	78	(Y = C or T)
	***Reverse***	GGGTAACATGTCTTCCATGCA		
	***Probe***	AGGGTCGCTGCCTACACTGCTG		
**HPV 11**	***Forward***	GCTTCATAAAACTAAATAACCAGTGG	110	
	***Reverse***	CAGGAGGCTGCAGGTCTAG		
	***Probe***	CCAGCAGTGTAAGCAACGACCC		
**HPV 16**	***Forward***	GCAGATCATCAAGAACACGTAG	108	
	***Reverse***	TAGAGATCAGTTGTCTCTGGTTG		
	***Probe***	CATGCATGGAGATACACCTACATTGCATG		
**HPV 18**	***Forward***	AGAGGCCAGTGCCATTCG	65	
	***Reverse***	GTTTCTCTGCGTCGTTGGAG		
	***Probe***	CTGTCGTGCTCGGTTGCAGC		
**HPV 33**	***Forward***	TTTCGGGTCGTTGGGCA	71	
	***Reverse***	ACGTCACAGTGCAGTTTCTC		
	***Probe***	CCTCCAACACGCCGCACA		
**HPV 45**	***Forward***	CAGTACCGAGGGCAGTGTA	68	
	***Reverse***	TCCCTACGTCTGCGAAGTC		
	***Probe***	CATGTTGTGACCAGGCACGGC		

### ddPCR experiments

1× ddPCR Supermix (Bio-Rad, USA), 1.0 µM primer, 0.25 µM probe, and 5 µL sample DNA were prepared into a 20 µL reaction liquid, thoroughly mixed, and transferred to a DG8 Cartridge. Next, droplet generation oil for probes was added to the bottom row of the DG8 Cartridge at (70 µL /hole), which was placed into the QX200 Droplet Generator™ (Bio-Rad). Droplets are generated in the top row of the DG8 cartridges. Thereafter, the generated droplets were carefully transferred to a 96-well PCR plate and sealed using the preheated PX1 plate sealer (Bio-Rad) for 175℃, 3.5 s. Subsequently, the sealed plate was placed in a thermal cycler (Bio-Rad), with the cycle conditions being as follows: the enzyme was activated at 95^◦^C for 10 min, followed by 94^◦^C for 30 s, 70℃ for 20 s, 59.5℃ for 1 min for 4 cycles, followed by 4℃ 30 s, 70℃ 20s, 85℃ 30s, 70℃ 20s, 59.5℃ 1 min for another 36 cycles. The enzyme was then deactivated at 98℃ (10 min), and the reaction was kept at 4℃. The ramp rate of ≤ 2.5℃/s was maintained during the whole process. Following PCR, the 96-well PCR plate was placed in the QX200 Droplet reader™ (Bio-Rad) and the ddPCR data was analyzed using the QuantaSoft analysis tool.

### Accuracy, specificity, sensitivity, repeatability and reproducibility evaluation of ddPCR

To evaluate the accuracy of ddPCR, positive samples with a low viral load for each HPV genotype as determined by HPV genotyping were selected. For sensitivity evaluation, high concentrations of HPV DNA of different genotypes were diluted from 10^5^ to 10^0^ copies/µL, and each concentration was tested three times. To evaluate specificity, two different negative controls were used: a human HPV-negative control and a pooled M-PC. To evaluate the repeatability of ddPCR, serial dilutions were tested in triplicate in one experimental run and in three independent experiments to assess reproducibility.

### Statistical analysis

Droplet reader software results were represented as copies/µL for each target (HPV genotype and control gene). For method optimization, intra-assay variability (repeatability) and inter-assay variability (reproducibility) were evaluated by calculating the coefficient of variation (CV). SPSS software (version 24.0; IBM Corp., Chicago, IL, USA) was used for descriptive statistics. Pearson’s correlation tests were used to compare the accuracy and consistency of the ddPCR and qPCR analyses. P < 0.05 was considered to indicate a statistically significant difference.

## Results

### Study cohort

**Fig. 1:**
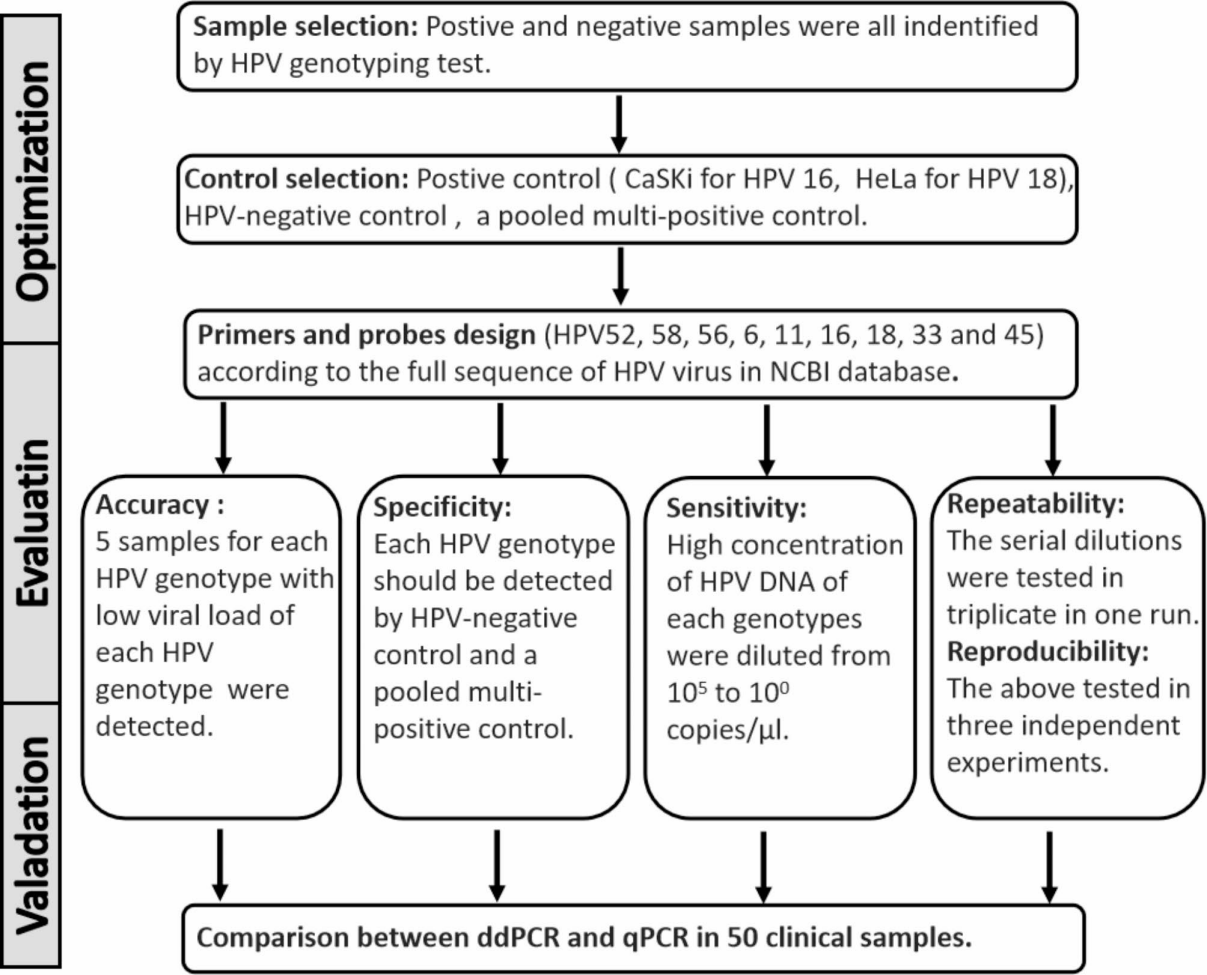
**Study cohorts.** Primers and probes designing; accuracy, specificity, sensitivity, repeatability and reproducibility evaluating; Clinal sample verification

Given the specificity of HPV infection in the Chinese population, primers and probes for HPV52, 58, 56, 6, 11, 16, 18, 33, and 45 were designed (Table 1). Next, the accuracy, specificity, sensitivity, repeatability, and reproducibility of ddPCR were evaluated. Finally, a comparative analysis between ddPCR and qPCR was performed using clinical samples (Fig. 1).

### Development of HPV ddPCR assays

Because the selected samples have a low HPV viral load as determined by HPV genotyping, the amplification signal of ddPCR is not very strong. For accuracy analysis, all HPV genotype assays yield positive droplets with channel amplitude signals between 3000 and 14,000, whereas different genotypes have different channel amplitude signals. By doing so, the viral load in 20 µL of input total DNA ranged from 7.2 to 318 copies in the samples positive for HPV52, 16 to 2,874 copies for HPV58, 12 to 654 copies for HPV52, 3.8 to 36 copies for HPV6, 5.2 to 28 copies for HPV11, 3.2 to 1358 copies for HPV16, 1.4 to 247.4 copies for HPV18, 3.8 to 496 copies for HPV33, and 11.2 to 324 copies for HPV45 (Table 2).

**Table 2:** The quantification of positive HPV samples with low viral load (high Ct values) detected by ddPCR

	Range of viral load by ddPCR (copy/ul)			Range of qPCR quantification (Ct value)	
	**High**	**Low**		**High**	**Low**
**HPV 52**	15.90	0.36		29.17	36.21
**HPV 58**	143.70	0.80		25.43	33.61
**HPV 56**	32.70	0.60		28.04	34.25
**HPV 6**	1.80	0.19		31.32	35.61
**HPV 11**	1.40	0.26		32.18	36.51
**HPV 16**	67.90	0.16		27.23	36.62
**HPV 18**	12.37	0.07		31.45	37.83
**HPV 33**	24.80	0.19		30.28	35.23
**HPV 45**	16.20	0.56		28.64	34.29

### Evaluation and comparison of ddPCR and qPCR in clinical samples

**Fig. 2:**
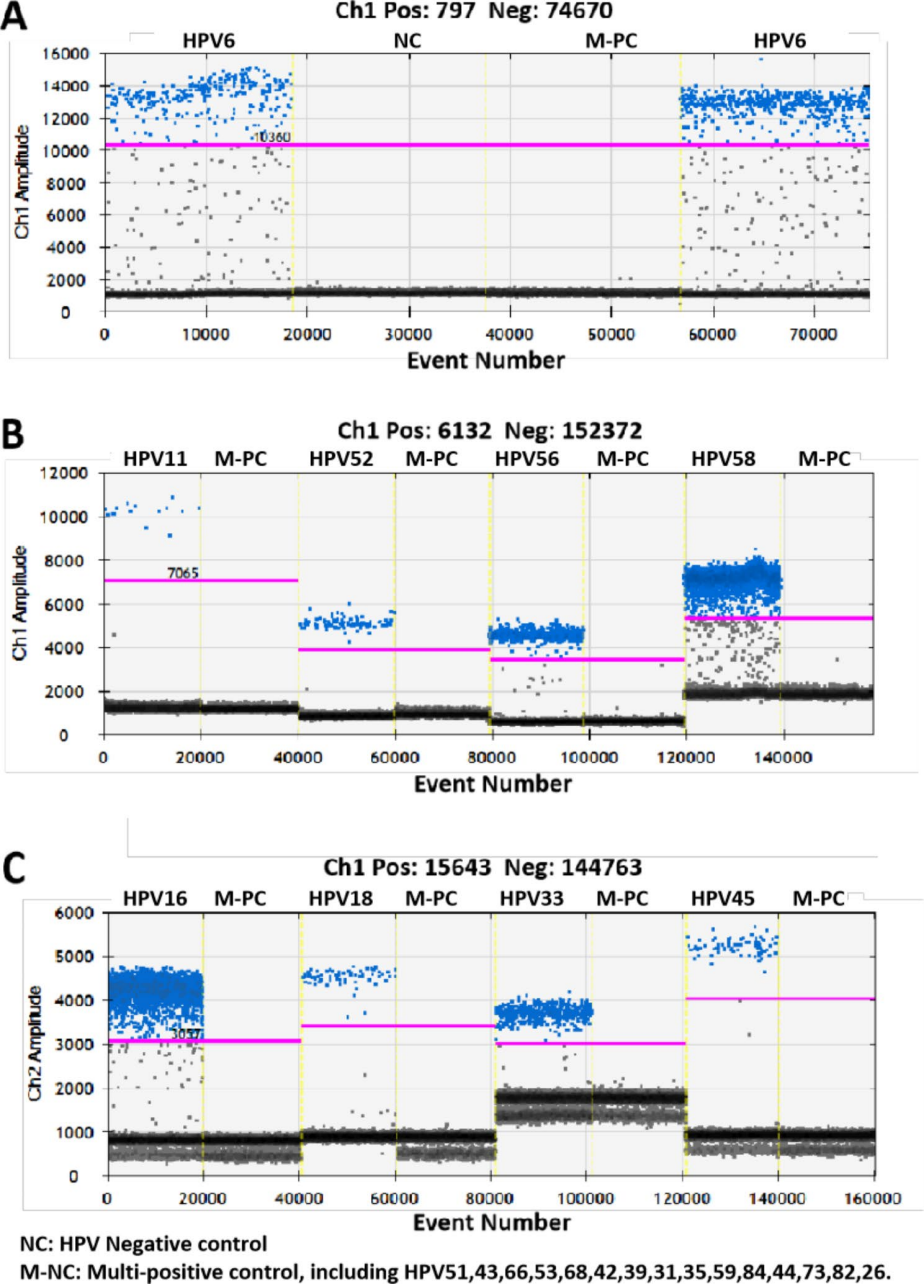
**Digital droplet PCR (ddPCR) for the detection of HPV 6, 11, 52, 56, 58, 16, 18, 33, 45.** Vertical lines represent the fluorescent amplitude of different HPV genotypes. Two negative controls are included: one is from negative sample, whereas the additional negative control is a mixed positive samples including other HPV genotypes. Droplets positive (blue), Droplets negative (gray)

**Fig. 3:**
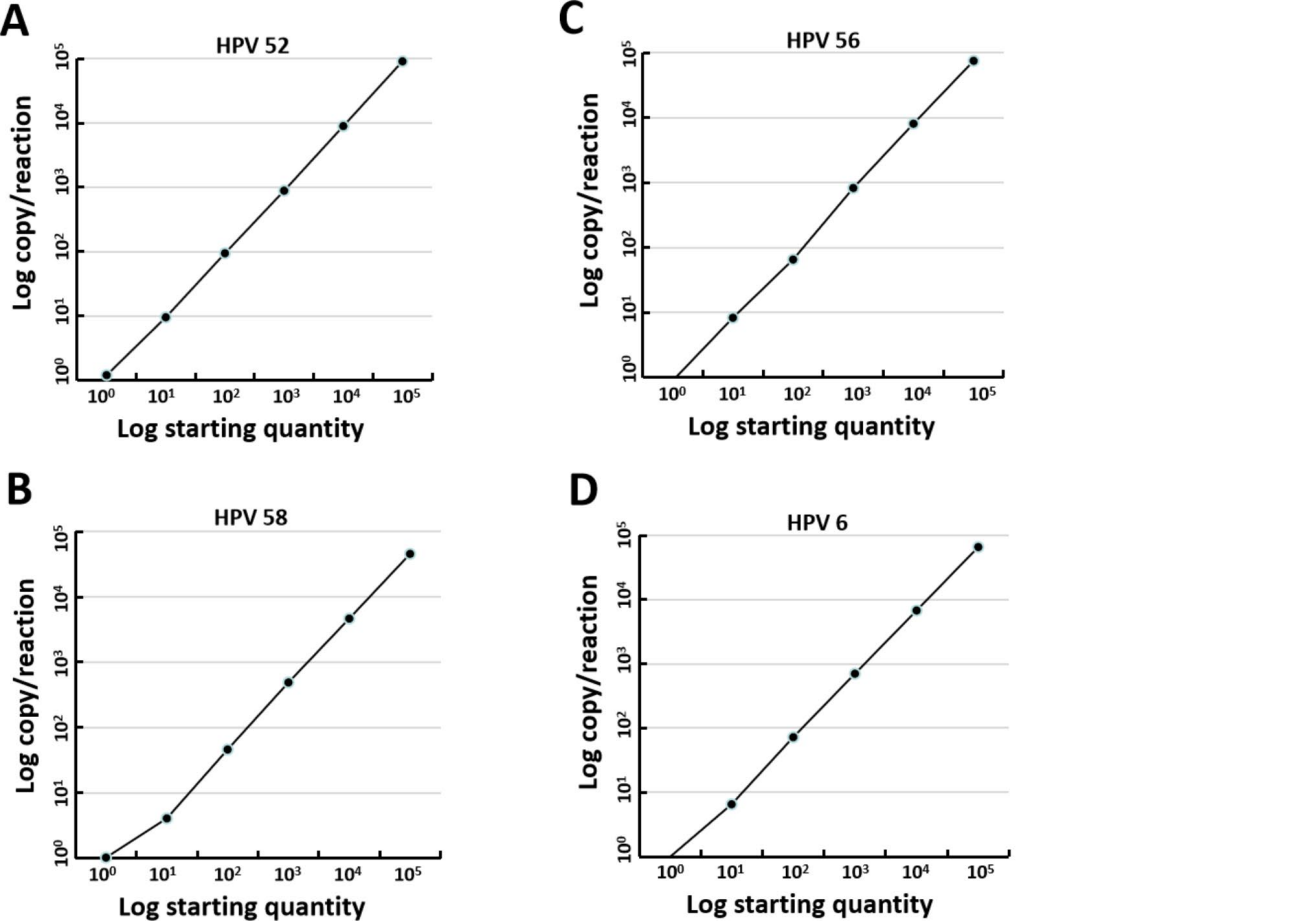
**The correlation between theoretical and detected plasmid concentrations of HPV 52, 56, 58, 6 by ddPCR.** Log copy/reaction against the log starting concentration of 10-fold serial dilutions were assessed. Horizontal lines represent the different plasmid concentrations: 10^0^, 10^1^, 10^2^, 10^3^, 10^4^, 10^5^ copies/µL, and the vertical lines represent the actual detected concentration

In the specificity analysis, all HPV genotype assays yielded no amplification signal in the HPV-negative control and a pooled multi-positive control (Fig. 2), indicating that no cross-reactivity occurred. To assess the assay sensitivity, samples containing certain HPV genotypes with DNA concentrations ≥ 10^5^ copy/µL were selected and then diluted 10-fold to 10^− 1^ copy/µL. Each dilution was tested by ddPCR in triplicate in one experiment run, and the result indicated a good degree of linear correlation with the dilutions (except for 10^− 1^ copy number/reaction, which could not be detected) of all tested HPV genotypes (Fig. 3, Supplementary Fig. 1). The minimum viral load was calculated (limit of detection, LOD) to determine the sensitivity of each assay. For this, the positive samples of each genotype is diluted to 10^0^ copies/µL and the test is repeated 20 times; the obtained triple standard deviation is considered the LOD. By doing so, we found that the viral LOD in a 20 µL reaction mixture presented in Table 3, that is 1.2 copies of HPV52, 1.02 copies of HPV58, 0.89 copies of HPV56 and 0.99 copies of HPV6.

To evaluate repeatability and reproducibility, 10-fold serial dilutions were tested in three independent experiments using ddPCR. The intra- and inter-assay coefficients of variation (CV), the mean concentrations of viral load, and the standard deviations (SDs) are shown in Table 3 and Supplementary Tables 1, respectively. The intra-assay CV values ranged from 0.6 to 38.3% for HPV52, 1.1–42.2% for HPV58, 0.9–40.8% for HPV56, and 1–37.6% for HPV6. Like-wise, the inter-assay CV values ranged from 0.5 to 35.1% for HPV52, 1.3–41.7% for HPV58, 0.7–36.3% for HPV56, and 0.6–42.6% for HPV6. These results indicate that our ddPCR assay has repeatability and reproducibility, with high sensitivity, accuracy, and specificity for quantifying HPV DNA, especially under conditions of low template copy number in CIN and/or cervical cancer.

**Table 3:** Repeatability and reproducibility of ddPCR assay for HPV 52/56/58/6

HPV	Intra-assay variation				Inter-assay variation		
**Viral load**	**Mean**	**SD**	**CV**		**Mean**	**SD**	**CV**
**HPV 52**							
100,000	90800.00	523.07	0.6%		91257.00	468.20	0.51%
10,000	8945.07	126.50	1.41%		9045.32	201.01	2.22%
1000	884.50	24.70	2.79%		894.10	54.56	6.10%
100	94.20	13.24	14.06%		91.04	13.56	14.89%
10	9.51	2.87	30.18%		9.23	3.24	35.10%
1	1.06	0.46	38.33%		1.56	0.49	31.41%
**HPV 58**							
100,000	45440.00	498.20	1.10%		43931.00	560.30	1.28%
10,000	4652.08	301.40	6.48%		4856.24	183.20	3.77%
1000	489.42	39.68	8.11%		504.00	23.00	4.56%
100	46.05	8.30	18.02%		48.60	10.02	20.62%
10	4.06	1.18	29.06%		5.42	2.03	37.45%
1	0.97	0.43	42.16%		0.84	0.35	41.67%
**HPV 56**							
100,000	74875.00	654.30	0.87%		75032.10	553.00	0.74%
10,000	8054.40	241.20	2.99%		7956.42	162.30	2.04%
1000	823.32	35.62	4.33%		810.30	46.40	5.73%
100	86.24	18.37	21.30%		81.09	16.82	20.74%
10	8.28	3.38	40.82%		8.20	1.64	20.00%
1	1.13	0.33	37.08%		1.02	0.37	36.27%
**HPV 6**							
100,000	65824.50	656.40	1.00%		66587.40	428.00	0.64%
10,000	6784.00	182.30	2.69%		6689.10	94.04	1.41%
1000	704.21	46.01	6.53%		713.20	41.30	5.79%
100	72.33	12.34	17.06%		74.65	12.64	16.93%
10	6.54	2.46	37.61%		8.67	1.07	12.34%
1	0.89	0.32	32.32%		1.29	0.55	42.64%

To further prove the advantages of ddPCR, 50 clinical samples were analyzed using both ddPCR and qPCR (Table 4, and 5). Forty-two specimens from individuals who underwent routine health examinations were negative in both assays, whereas two additional samples that were positive for ddPCR tested negative in qPCR. Additionally, although four CIN samples were HPV-positive in both assays, ddPCR detected HPV DNA in two CIN samples that qPCR failed to detect. Our data are consistent with those of previous studies, showing that HPV16 is the most common genotype in cervical precancerous lesions, followed by HPV18 [16]. Calculated from the true positives and false positives as depicted in Table 4, the diagnostic sensitivity, specificity, and accuracy of ddPCR were 100.00%, 91.30%, and 92.00% respectively. In general, HPV DNA was detected in 8% (4/50) clinical samples with qPCR assay, whereas HPV DNA was detected in 16% (8/50) clinical samples with ddPCR assay, indicating a high detection rate for our ddPCR assay combined with qPCR.

**Table 4:** Detection results by ddPCR and qPCR in clinical samples

Sampels information			HPV genotypes	
**Number**	**Catogory**		**qPCR**	**ddPCR**
**1**	CC I A1			HPV16
**2**	CC II A1		HPV16	HPV16
**3**	CC I A1			HPV18
**4**	CC I A2		HPV16	HPV16
**5**	CC I B1		HPV16	HPV16
**6**	CC II B1		HPV18	HPV18
**7**	Routine physical examination specimens			HPV58
**8**				HPV16
**9–50**				

**Table 5:** Comparative analysis between ddPCR and qPCR in clinical samples

Method	ddPCR		
**qPCR**	**Postive**	**Negative**	**Total**
**Postive**	4	0	4
**Negative**	4	42	46
**Total**	8	42	50

## Discussion

HPV viral load is closely related to viral persistence [17], and different HPV genotypes have different consequences. The risk of continuous progression can be determined by identifying the HPV genotype and viral load [18]. Currently, the screening and diagnosis of cervical cancer include cytological examination, imaging modalities, and HPV detection [19]. However, the first two methods depend on the doctor’s ability to analyze the results [20] and are associated with poor repeatability, sensitivity, and long processing times [21–23].

qPCR obtains amplification times directly proportional to the fluorescent dye by amplifying DNA and comparing it with the standard sample to quantify the sample, which causes the results of qPCR to be affected easily by the standard curve, inhibitor, or background DNA [24, 25]. However, this method is not appropriate when the viral load is lower than the conventional detection limit at early or late stages [26, 27]. Low viral load measurements of HPV DNA may be an indicator of CIN onset/progression [10]. Moreover, it takes approximately ten years for HPV infections to develop into invasive cancers. Therefore, an accurate assessment of HPV viral load is an effective way to detect and evaluate cervical disease progression.

ddPCR is a new method based on quantitative PCR (qPCR) [24] that determines the absolute quantity of DNA by dispersing DNA in a large number of droplets, counting the number of positive and negative droplets, and detecting samples with lower DNA content [28]. The application of ddPCR in viral diseases, such as viral infection [29], microRNA analysis, genome editing, and detection, is becoming more and more extensive [30, 31], and gene copy number variation analysis, involves the detection of target nucleic acid [32–34]. Studies have reported the quantitative detection of HPV DNA using the ddPCR method [35–37]. Although the sensitivity and specificity of these studies are high, they have mainly focused on the identification of country-specific HPV genotypes, ignoring the infection specificity of HPV in different regions.

Based on existing studies on HPV16, 18, 33, 45, and 11, the current study was designed to supplement and evaluate ddPCR for viral load quantification of HPV genotypes 52, 56, 58, and 6 in the Chinese population. To avoid overtreatment at the early stage of HPV infection, our ddPCR method is more applicable to clinical samples at pre-cancerous stages, especially when the template copy number is low in CIN and/or cervical cancer. Previous PCR/qPCR primers detected additional HPVs [38]. To avoid this potential risk, a multi-positive control named M-PC, including HPV51, 43, 66, 53, 68, 42, 39, 31, 35, 59, 84, 44, 73, 82, and 26, was used, and the results showed that our ddPCR primers could not be extended for the detection of additional HPVs. Additionally, HPV can be detected in the blood in the form of a free body or integrated into tumor DNA [39–41], and some studies have investigated circulating tumor DNA, microRNA, and/or viral DNA/miRNA using ddPCR in gynecologic cancer [42, 43]. In the future, based on the infection specificity in the Chinese population, we plan to use noninvasive samples, such as saliva and blood samples, instead of cervical lesions for ddPCR detection and optimization.

In conclusion, the ddPCR method used in this study exhibited high sensitivity, accuracy, and specificity in quantifying HPV DNA sequences. The technique may be used as a promising assay for the early detection of cervical cancer and may help to evaluate treatment response and timely monitoring of the disease to prevent overtreatment.

### Electronic supplementary material

Below is the link to the electronic supplementary material.

Supplementary Material 1

Supplementary Material 2

## Data Availability

All datasets generated for this study are included in the article.
