# Supplementary material for: Development of a sensitive droplet digital PCR according to the HPV infection specificity in Chinese population
Source: BMC Cancer. 2023 Oct 23;23:1022. doi: 10.1186/s12885-023-11529-3 (PMC10594741; doi:10.1186/s12885-023-11529-3)
Supplement: Supplementary file 1 — Supplementary Material 1 [file 12885_2023_11529_MOESM1_ESM.doc]

| **Supplemental table 1** | | | | | | | |
| --- | --- | --- | --- | --- | --- | --- | --- |
| **Repeatability and reproducibility of ddPCR assay for HPV 16/18/33/45/11.** | | | | | | | |
| **HPV** | **Intra-assay variation** | | |  | **Inter-assay variation** | | |
| **Viral load** | **Mean** | **SD** | **CV** |  | **Mean** | **SD** | **CV** |
| **HPV 16** |  |  |  |  |  |  |  |
| 100000 | 92456.00 | 1206.41 | 1.30% |  | 90837.63 | 1235.93 | 1.36% |
| 10000 | 9454.20 | 112.97 | 1.19% |  | 9061.33 | 85.95 | 0.95% |
| 1000 | 995.10 | 28.20 | 2.83% |  | 1005.50 | 21.28 | 2.12% |
| 100 | 102.20 | 10.78 | 10.55% |  | 989.20 | 17.16 | 1.73% |
| 10 | 10.29 | 0.77 | 7.52% |  | 9.83 | 0.50 | 5.06% |
| 1 | 1.36 | 0.26 | 19.45% |  | 1.08 | 0.14 | 12.51% |
| **HPV 18** |  |  |  |  |  |  |  |
| 100000 | 100705.77 | 804.41 | 0.80% |  | 100124.77 | 315.21 | 0.31% |
| 10000 | 10063.17 | 79.20 | 0.79% |  | 10013.83 | 85.32 | 0.85% |
| 1000 | 1025.27 | 28.01 | 2.73% |  | 1011.60 | 27.95 | 2.76% |
| 100 | 100.88 | 4.94 | 4.90% |  | 93.88 | 0.37 | 0.39% |
| 10 | 10.47 | 0.70 | 6.71% |  | 9.41 | 0.60 | 6.35% |
| 1 | 1.05 | 0.17 | 16.18% |  | 0.79 | 0.18 | 22.39% |
| **HPV 33** |  |  |  |  |  |  |  |
| 100000 | 96287.30 | 1893.77 | 1.97% |  | 91154.63 | 1886.38 | 2.07% |
| 10000 | 9583.87 | 529.79 | 5.53% |  | 9217.00 | 331.14 | 3.59% |
| 1000 | 981.77 | 69.84 | 7.11% |  | 1035.17 | 81.19 | 7.84% |
| 100 | 103.87 | 5.49 | 5.28% |  | 100.87 | 3.37 | 3.34% |
| 10 | 10.35 | 0.49 | 4.70% |  | 11.16 | 1.57 | 14.07% |
| 1 | 1.23 | 0.15 | 12.48% |  | 1.00 | 0.13 | 12.55% |
| **HPV 45** |  |  |  |  |  |  |  |
| 100000 | 51290.47 | 949.28 | 1.85% |  | 50409.47 | 1487.73 | 2.95% |
| 10000 | 5336.60 | 170.23 | 3.19% |  | 4949.60 | 76.45 | 1.54% |
| 1000 | 541.57 | 27.18 | 5.02% |  | 463.37 | 20.34 | 4.39% |
| 100 | 55.87 | 2.20 | 3.93% |  | 53.53 | 5.18 | 9.67% |
| 10 | 5.31 | 0.47 | 8.91% |  | 4.53 | 0.27 | 5.87% |
| 1 | 0.37 | 0.04 | 11.15% |  | 0.53 | 0.09 | 16.17% |
| **HPV 11** |  |  |  |  |  |  |  |
| 100000 | 99200.17 | 184.54 | 0.19% |  | 100135.17 | 189.26 | 0.19% |
| 10000 | 9893.97 | 306.48 | 3.10% |  | 10134.90 | 401.81 | 3.96% |
| 1000 | 990.27 | 14.85 | 1.50% |  | 1044.83 | 42.15 | 4.03% |
| 100 | 99.33 | 1.05 | 1.05% |  | 105.63 | 4.76 | 4.51% |
| 10 | 10.35 | 0.24 | 2.35% |  | 10.42 | 0.22 | 2.08% |
| 1 | 1.12 | 0.08 | 6.87% |  | 1.09 | 0.09 | 8.30% |
