# Supplementary material for: Development of a sensitive droplet digital PCR according to the HPV infection specificity in Chinese population
Source: BMC Cancer. 2023 Oct 23;23:1022. doi: 10.1186/s12885-023-11529-3 (PMC10594741; doi:10.1186/s12885-023-11529-3)
Supplement: Supplementary file 2 — Supplementary Material 2 [file 12885_2023_11529_MOESM2_ESM.doc]

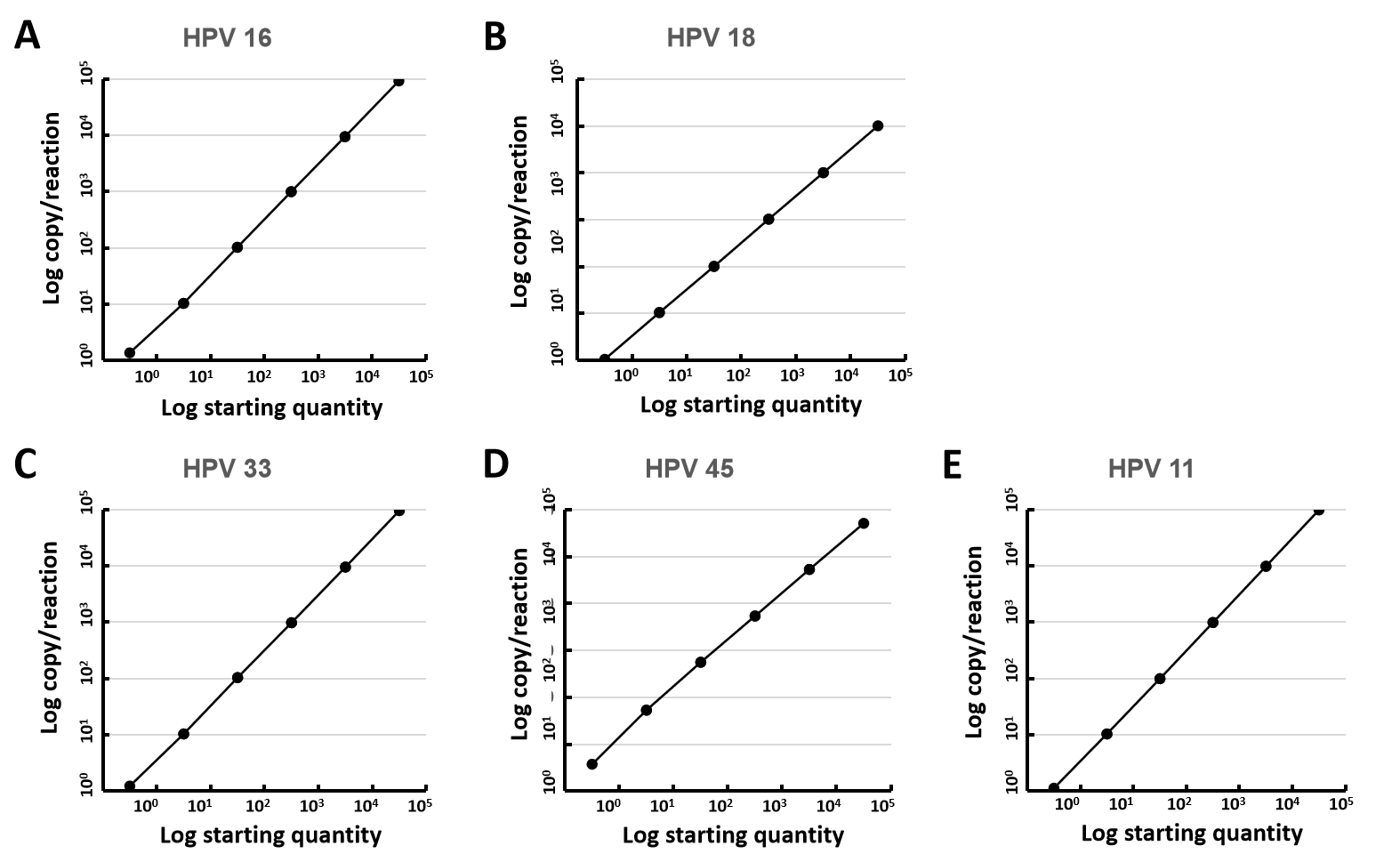


**Supplemental figure1.** **The correlation between theoretical and detected plasmid concentrations of HPV16, 18, 33, 45, 11 by ddPCR.** Log copy/reaction against the log starting concentration of 10-fold serial dilutions were assessed. Horizontal lines represent the different plasmid concentrations: 10^0^, 10^1^, 10^2^, 10^3^, 10^4^, 10^5^ copies/µL, and the vertical lines represent the actual detected concentration.
